# Genome Sequence of the Octopine-Type *Agrobacterium tumefaciens* Strain Ach5

**DOI:** 10.1128/genomeA.00225-14

**Published:** 2014-03-27

**Authors:** Christiaan V. Henkel, Amke den Dulk-Ras, Xiaorong Zhang, Paul J. J. Hooykaas

**Affiliations:** Department of Molecular and Developmental Genetics, Institute of Biology, Leiden University, Leiden, The Netherlands

## Abstract

We have sequenced the complete genome of the plant pathogen *Agrobacterium tumefaciens* strain LBA4213, a derivative of the wild-type strain *A. tumefaciens* Ach5 and the ancestor of *A. tumefaciens* strain LBA4404 used in genetic engineering. The genome consists of a circular chromosome and a linear chromosome, as well as a megaplasmid and a tumor-inducing plasmid.

## GENOME ANNOUNCEMENT

Soil bacteria of the species *Agrobacterium tumefaciens* are the causative agents of crown gall disease in plants. They induce plant tumors by transferring genes encoding phytohormones and opine biosynthetic enzymes from a tumor-inducing (Ti) plasmid to plant cell genomes.

*A. tumefaciens* strain Ach5 belongs to the tumor-inducing biotype I class of agrobacteria and harbors a Ti plasmid of the octopine type. *A. tumefaciens* strain LBA4213 is a Tn*904*-mutagenized derivative of the wild-type Ach5 (1, 2). LBA4213 is itself the immediate precursor of *A. tumefaciens* strain LBA4404, which has a major biotechnological application as the genomic host for the binary vector system employed in the genetic engineering of plants (3). *A. tumefaciens* LBA4404 differs from LBA4213 only by the deletion of a large region of the Ti plasmid.

The genome of *A. tumefaciens* strain LBA4213 was sequenced using 99-nucleotide paired-end reads on an Illumina HiSeq 2000. A total of 7,098,396 read pairs were obtained (approximately 250× coverage). Sequencing errors were removed using Quake version 0.3 (4), and the reads were assembled into 99 contigs, with an *N*_50_ of 585,488 bp, using Velvet version 1.2.03 (5).

Major ambiguous connections in the assembly graph were resolved using targeted PCRs. At minor ambiguities (e.g., variation within repeats), nucleotides were masked by Ns (1,365 positions in total). Full-length chromosome and plasmid sequences were reconstructed from the graph by making reasonable assumptions about the topology of individual molecules (e.g., the existence of a circular Ti plasmid). The final genome assembly is 5.63 Mb in length, has a C+G content of 58.5%, and consists of a circular chromosome of 2,773,134 bp, a linear chromosome of 2,095,074 bp, a “cryptic” megaplasmid of 556,485 bp, and a Ti plasmid of 205,997 bp. More than 99.95% of the corrected reads align to these sequences, and apart from masked sequence, every position is covered, indicating the assembly is complete.

Finished sequences were submitted to the NMPDR RAST server (version 4.0) for the annotation of protein-coding, rRNA, and tRNA genes (6). Gene predictions for the virulence genes on the Ti plasmid were validated manually using the previously annotated octopine Ti plasmid sequence (7). In total, 5,432 protein-coding sequences were annotated, as well as 5 clusters of rRNA genes and 52 tRNA genes. Both chromosomes contain rRNA and tRNA genes, whereas the plasmids do not.

Based on its *recA* housekeeping gene variant, LBA4213 is a member of genomospecies 1 of *Agrobacterium* biotype I (8). To date, several *Agrobacterium* genome sequences have been finalized, revealing a division of the chromosomes into a linear molecule and a circular molecule unique to biotype I (9–11). Both chromosomes of LBA4213 are mostly collinear with the previously published complete chromosomes of *A. tumefaciens* strain C58 and *Agrobacterium* sp. strain H13-3. However, the megaplasmid shows little conservation between the three sequenced species, with only 12.4% and 26.6% aligning to C58 and H13-3 counterparts, respectively. Except for the Tn*904* insertion encoding streptomycin resistance, the Ti plasmid is 99.8% identical to the previously published octopine-type consensus (7).

### Nucleotide sequence accession numbers.

The complete genome sequence of *A. tumefaciens* LBA4213 has been deposited in GenBank under accession no. CP007225 to CP007228.
